# Biodegradable Mg–Mo_2_C MXene Air Batteries for Transient Energy Storage

**DOI:** 10.1021/acsami.3c17692

**Published:** 2024-03-18

**Authors:** Shunsuke Yamada

**Affiliations:** Department of Robotics, Tohoku University, Room 113, Building No. A15, Area A01, 6-6-01 Aoba, Aramakiaza, Aobaku, Sendaishi, Miyagi 980-8579, Japan

**Keywords:** MXene, primary battery, ionic liquid, transient electronics, nanomaterial

## Abstract

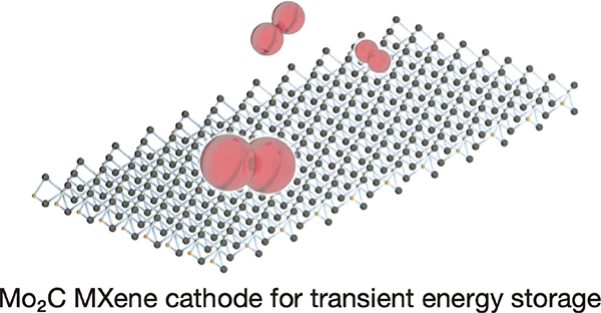

Primary batteries are the fundamental power sources in small electronic gadgets and bio/ecoresorbable batteries. They are fabricated from benign and biodegradable materials and are of interest in environmental sensing and implants because of their low toxicity toward the environment and human body during decomposition. However, current bio/ecoresorbable batteries suffer from low operating voltages and output powers because of the occurrence of undesired hydrogen evolution reactions (HERs) at cathodes. Herein, Mo_2_C MXene was used as a cathode to achieve high operating voltage and areal power. Mo_2_C provides energy barriers for HERs in alkaline solutions, and such barriers suppress HERs and allow the oxygen reduction reaction to dominate at the cathode. The fabricated battery exhibits an operating voltage and areal power of 1.4 V and 0.92 mW cm^–2^, respectively. Degradation tests show that the full cell completely degrades within 123 days, leaving only Mo fragments from the electrode and biodegradable encapsulation. This study provides insights into bio/ecoresorbable batteries with high power and operating voltage, which can be used for environmental sensing.

## 1 Introduction

With the development of electronics, the applications of wireless sensor modules have expanded from indoors to outdoors and even inside humans (e.g., implants). Because electronic devices are used outdoors and as implants, their retrieval after use is an inevitable issue that needs to be resolved to avoid environmental pollution and adverse effects on human health. Therefore, devices that disappear without leaching harmful substances are highly desired. Transient electronics^1^ have attracted considerable attention with biodegradable materials^2−4^ in environmental sensing, implants, and wearable devices due to their high biodegradability, biocompatibility, and environmental benignity, and researchers have developed transient electronic devices such as transistors,^5−7^ sensors,^8−12^ and photonic devices.^13−15^ Transient energy storage is essential to operate electronic devices; therefore, transient supercapacitors,^16−18^ pseudocapacitors,^19−21^ and batteries^22−28^ have also been developed. Transient primary batteries have been studied because of their facile fabrication, high energy capacity, and use of benign materials. These batteries incorporate metals with low (e.g., magnesium (Mg) or zinc (Zn)) and high (e.g., iron (Fe), molybdenum (Mo), or tungsten (W)) standard hydrogen electrode (SHE) potentials as anodes and cathodes, respectively. Mg–X (X = Fe, Mo, or W) batteries exhibit Mg corrosion reactions and hydrogen evolution reactions (HERs) at the anode and cathode, respectively, the equations of which are written as follows:^22^Mg corrosion:
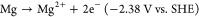
1and the HER:
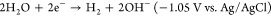
2

The following oxygen reduction reaction (ORR) simultaneously occurs at the cathode:
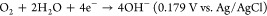
3

The cathodic potential ranges from −1.05 to 0.179 V vs Ag/AgCl, which affords a small output voltage of ≈0.8 V. To increase the operating voltages of bio/ecoresorbable batteries, Jia et al. used Au as a cathode to develop Mg–Au air batteries, where Au cathodes hinder the HER, making the ORR dominant. The large difference between the anodic and cathodic potentials of Mg–Au air batteries affords an output voltage of 1.6 V.^23^ Huang et al. and Karami-Mosammam et al. developed Mg–MoO_3_ batteries by coating Mo cathodes with MoO_3_. MoO_3_ can accommodate small ions in its interlayers, and this reaction is described as the intercalation of multivalent (^*n*+^) metal ions (M^*n*+^),^29,30^ as follows:
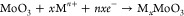
4

This reaction has a larger reduction potential than the HER potential, which affords an operating voltage of 1.5 V. Huang et al. used iodine I_2_ as cathode to fabricate Mg–I batteries, which undergo reduction reactions as follows:
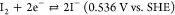
5

Moreover, fabricated Mg–I batteries exhibit a high operating voltage of ≈1.8 V^24^.

Mg–X and Zn–Mo batteries exhibit excellent biodegradability but suffer from a low operating voltage of <0.8 V.^31^ In Mg–Au air batteries, Au, a precious metal, is used; however, Au is not suitable for fabricating batteries with enormous wireless sensor modules. Carbon black was dispersed in I_2_ to increase electrical conductivity and minimize I_2_ leaching. C and Au are stable and remain in the environment and human bodies after the decomposition of batteries. Although Mg–MoO_3_ batteries completely biodegrade during in vivo and in vitro tests, they suffer from a low power density of 0.27 mW cm^–2^, which is the maximum obtained. Such low power density cannot operate electronic devices with high power consumption.

**Figure 1:**
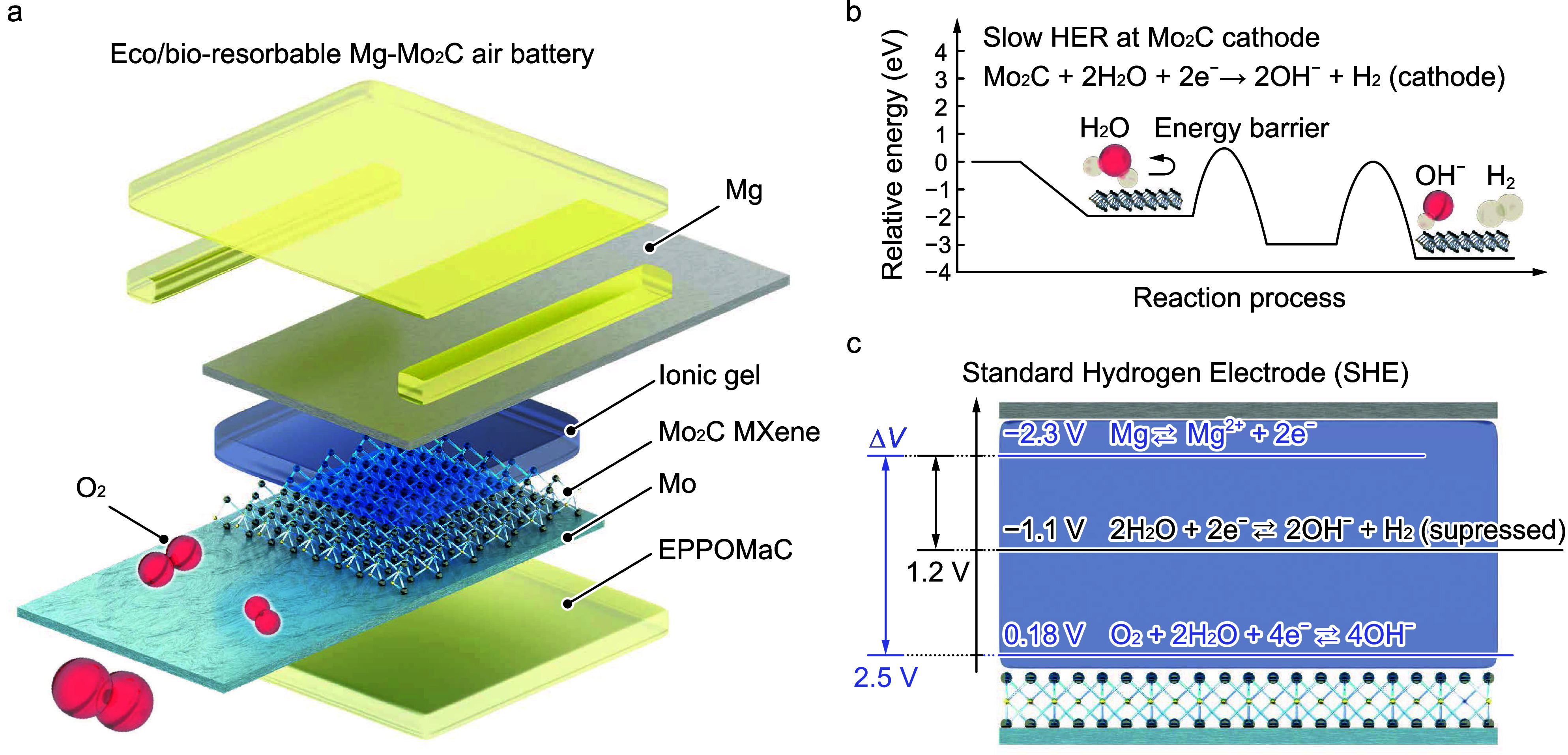
Schematic illustration and operation principle of the battery. (a) Device configuration of the battery showing that it used Mg, Mo_2_C, Mo foil, IG, and EPPOMaC as the anode, cathode, current collector, electrolyte, and substrate/spacer, respectively. (b) Energy diagram of the hydrogen evolution reaction (HER) at the Mo_2_C cathode in an alkaline solution. (c) Energy barriers hinder the HER, and the main reaction in the battery is the Mg oxidization and oxygen reduction reaction (ORR) at the anode and cathode, respectively, which yield an ideal operating voltage of 2.5 V.

Herein, a biodegradable air battery with Mo_2_C (MXene) cathodes was fabricated to address the issues of low operating voltage and power density. Biodegradable materials, i.e., Mg,^5^ Mo_2_C,^32^ Mo,^33^ ionic gels (IGs),^21^ and ester bond cross-linked photo-cross-linked poly(octamethylene maleate (anhydride) citrate) (EPPOMaC),^11^ were used as the anode, cathode, cathodic current collector, electrolyte, and substrate/spacer, respectively, as shown in Figure 1a. An ionic liquid (IL), [Ch][Lac], was produced using bioderived materials, i.e., choline and lactate, and exhibited ready biodegradability.^34^ [Ch][Lac] was dispersed in poly(vinyl alcohol) (PVA) to prepare the IG. MXenes are an emerging class of two-dimensional materials and exploited for energy storage devices due to their high electrical conductivity,^35,36^ large surface areas,^37,38^ and inhibition of the dendrite growth at anodes.^39,40^ Furthermore, Mo_2_C exhibited slow HER kinetics in alkaline solutions due to its energy barriers, as shown in Figure 1b.^41^ Because of these energy barriers, Mg–Mo_2_C batteries suppressed the HER and exhibited a dominant ORR in the cell, which increased the potential difference between the anode and cathode and exhibited an ideal operating voltage of 2.5 V, as shown in Figure 1c. A Mo_2_C cathode (Mo_2_C_1.0g_) was prepared using 1.0 g of delaminated Mo_2_CT_*x*_ (d-Mo_2_C), and Mg, Mo_2_C, and IG were used to make a battery (Mg–Mo_2_C_1.0g_–IG). Fabricated Mg–Mo_2_C_1.0g_–IG batteries exhibited an open circuit potential and operating voltages of 1.6 and 1.4 V at 0.1 mA cm^–2^, respectively. The discharge characteristics indicated that the capacity and maximum power of the Mg–Mo_2_C_1.0g_–IG batteries were 0.57 mAh cm^–2^ and 0.92 mW cm^–2^, respectively. The serially connected Mg–Mo_2_C_1.0 g_–IG 2 cells exhibited an operating voltage of 2.8 V, and they could be used to power a wireless sensor module to realize wireless sensing. Dissolution tests indicated that individual components, i.e., Mg and Mo_2_C_1.0g_, dissolved in 100 mL of phosphate-buffered saline (PBS) at 37 °C after 39 and 89 days, respectively, whereas EPPOMaC did not dissolve in PBS but its mass decreased by 20%. The Mg–Mo_2_C_1.0g_–IG 1 cell dissolved completely within 123 days, leaving Mo fragments and EPPOMaC. This biodegradable primary battery offers an opportunity to develop wireless sensing systems that can be used in environmentally benign devices, sustainable electronics, and bioresorbable/implantable devices.

## 2 Results and Discussion

### 2.1 MXene Synthesis and Characterization

**Figure 2:**
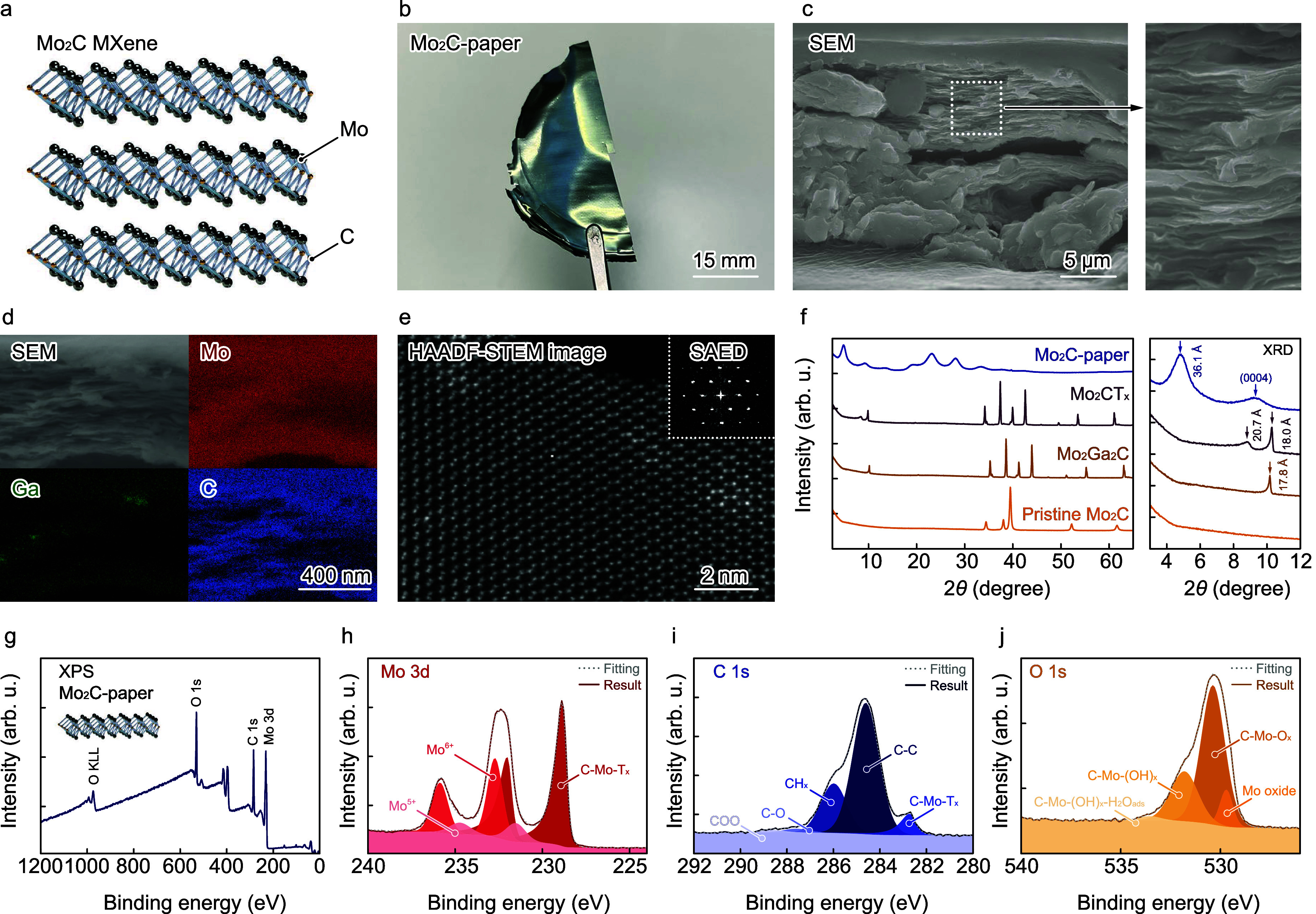
Synthesis and characterization of Mo_2_C MXene. (a) Schematic and (b) photograph of Mo_2_C paper. (c) Cross-sectional SEM image of Mo_2_C paper (left) and magnified image of the laminated nanosheets (right). (d) SEM image of the Mo_2_C paper and its corresponding EDX mapping of Mo, Ga, and C. (e) HAADF-STEM image of monolayer Mo_2_C; the inset shows its SAED pattern. (f) XRD patterns of pristine Mo_2_C, Mo_2_Ga_2_C, Mo_2_CT_*x*_, and Mo_2_C paper and the corresponding magnified patterns in the 2θ range from 3 to 12°. (g) Survey and core-level deconvoluted (h) Mo 3d, (i) C 1s, and (j) O 1s XPS spectra of the Mo_2_C paper.

As shown in Figure 1a, delaminated d-Mo_2_C layers were stacked to form Mo_2_C paper for the cathode of batteries. A previously reported method with some modifications was used to prepare Mo_2_C papers,^32,42−44^ and the details of the protocols are provided in Section 4. Briefly, pristine Mo_2_C powders and Ga flakes were homogeneously mixed using an agate mortar and vacuum annealed at 850 °C for 48 h to obtain an Mo_2_Ga_2_C powder. The Mo_2_Ga_2_C powder was soaked in a HCl solution to remove unreacted Ga. Mo_2_Ga_2_C is composed of the MAX phase, where M = transition metal, A = group 13 or 14 element, and X = carbon, as shown in Figure S1a, Supporting Information. Scanning electron microscopy (SEM) images show that the Mo_2_Ga_2_C powder has a flat flaky morphology with a size of <5 μm (Figure S1b, Supporting Information), and it has a layered structure (Figure S1c, Supporting Information). High-angle annular dark-field scanning transmission electron microscopy (HAADF-STEM) images show that alternating atomic double layers composed of heavy- and lightweight atoms are present in the Mo_2_Ga_2_C powder, as shown in Figure S1d, Supporting Information. The HAADF-STEM image (Figure S1e, Supporting Information) and corresponding energy-dispersive X-ray spectroscopy (EDX) maps of Mo (Figure S1f, Supporting Information), C (Figure S1g, Supporting Information), and Ga (Figure S1h, Supporting Information) show that the alternating layers of the Mo_2_Ga_2_C powder are composed of Mo and Ga. Although the sensitivity of EDX for C is low compared with that for Mo and Ga, C is present in the same layer as Mo in the Mo_2_Ga_2_C powder, as shown in the merged image (Figure S1i, Supporting Information). The Mo_2_Ga_2_C forms a hexagonal structure in the [0001] zone axis (Figure S2a,b, Supporting Information),^43,45^ and the HAADF-STEM images agree with the Mo_2_Ga_2_C crystal structure in the [112̅0] zone axis (Figure S2c, Supporting Information). The TEM observation and EDX confirm the synthesis of Mo_2_Ga_2_C with the MAX phase. 2.5 g of the Mo_2_Ga_2_C powder and 80 mL of a 49% hydrogen fluoride (HF) solution were sealed in a fluorinate container and heated at 55 °C for 160 h to remove the Ga layer from the MAX phase. The Mo_2_Ga_2_C powder without the Ga layer is henceforth named Mo_2_CT_*x*_. 1 g of the Mo_2_CT_*x*_ powder was dispersed in 4 mL of tetrabutylammonium hydroxide (TBAOH), followed by ultrasonication in an ice bath for 1 h to allow TBAOH to intercalate into the interlayers of Mo_2_CT_*x*_. Then, the Mo_2_CT_*x*_ powder was washed with ethanol and 1 g of the washed Mo_2_CT_*x*_ powder was dispersed in 4 mL of deionized water (DIW). Then, the dispersion solution was ultrasonicated and centrifuged to obtain a suspension of delaminated Mo_2_C (named d-Mo_2_C). An 8 mL portion of the d-Mo_2_C suspension was vacuum filtered onto nanoporous polypropylene membranes and dried to obtain dark-green Mo_2_C paper, as shown in Figure 2b. SEM images show that the thickness of Mo_2_C paper is ≈16 μm, and magnified SEM images show that MoC_2_ paper comprises d-Mo_2_C nanosheets (Figure 2c). The EDX mapping of Mo_2_C paper shows that most of the Ga was removed during HF etching (Figure 2d).

Figure 2e shows a HAADF-STEM image of a single sheet of d-Mo_2_C and its corresponding selected area electron diffraction (SAED) pattern. The HAADF-STEM captured the image in the [0001] direction, and a single sheet of d-Mo_2_C shows a hexagonal crystal structure. An annular bright-field (ABF)-STEM image conforms that C atoms exist at the center of a hexagonal structure in the HAADF-STEM image (Figure S3a, Supporting Information). Stacking of ABC and ABA atomic sequences forms 1T- and 2H-d-Mo_2_C,^46,47^ respectively, and the crystal structures of 1T-d-Mo_2_C in the [0001] and [112̅0] zone axes indicate that the MXene in this work possesses the 1T-d-Mo_2_C phase (Figure S3b,c, Supporting Information). The SAED pattern shows that d-Mo_2_C exhibits a highly crystallized structure without defects. X-ray diffraction (XRD) analysis was performed on pristine Mo_2_C, Mo_2_Ga_2_C, Mo_2_CT_*x*_, and Mo_2_C samples to determine their atomic structures. Figure 2f shows the XRD patterns of pristine Mo_2_C, Mo_2_Ga_2_C, Mo_2_CT_*x*_, and Mo_2_C paper in the 2θ range from 2.5 to 65° and from 3 to 12°. An intense peak at 9.9° is observed in the XRD pattern of Mo_2_Ga_2_C, corresponding to the MAX phase with a *c* lattice parameter of 17.8 Å, which is close to that reported in the literature.^32,42^ After Ga etching using an HF solution, a peak at 8.5° in addition to the 9.9° peak is observed in the XRD pattern of Mo_2_CT_*x*_. The *d* spacings of the 8.5 and 9.9° peaks are 20.8 and 18.0 Å, respectively. The expansion may be due to the intercalation of a monolayer of water molecules with a van der Waals radius of 2.8 Å.^42^ The XRD pattern of Mo_2_C paper shows a broad peak at 4.9°, corresponding to a *d* spacing of 36.1 Å. According to the literature, TBA cations remain in the interlayers of d-Mo_2_C during vacuum filtration and drying.^42^ X-ray photoelectron spectroscopy (XPS) was performed to analyze the surface states of the Mo_2_C paper. Figure 2g shows the survey XPS spectrum of Mo_2_C paper, and the core-level Mo 3d, C 1s, and O 1s XPS spectra of Mo_2_C paper are shown in Figure 2h, i, and j, respectively. As shown in Figure 2h, the intense peaks at 228.8 and 232.0 eV in the XPS spectrum of Mo_2_C paper are ascribed to Mo–C 3d_5/2_ and Mo–C 3d_3/2_ species, respectively.^42^ The deconvoluted spectrum of Mo_2_C paper shows that the peaks at 232.7 and 235.9 eV are attributed to the Mo 3d_5/2_ and Mo 3d_3/2_ spin–orbit components of the Mo^6+^ oxidation state, respectively. The minor peaks at 231.5 and 234.7 eV in the XPS spectrum of Mo_2_C paper correspond to Mo 3d_5/2_ and Mo 3d_3/2_ of the Mo^5+^ oxidation state, respectively. Mo_2_C paper was placed in ambient air, and its surface was oxidized. The deconvoluted core-level C 1s XPS spectrum of Mo_2_C paper exhibits five peaks at 282.7, 284.6, 286.0, 287.6, and 288.6 eV corresponding to C–Mo–T_*x*_, C–C, C–H_*x*_, C–O, and COO species, respectively.^42^ In the XPS spectrum of Mo_2_C paper, all peaks except the Mo–C peak are attributed to intercalated TBAOH molecules and/or exposure of Mo_2_C paper to the ambient air during storage.^42^ Furthermore, in the O 1s XPS spectrum of Mo_2_C paper, the deconvoluted peak at 529.7 eV proves the presence of Mo oxide. The Mo 3d XPS spectrum shows the presence of Mo^5+^ and Mo^6+^ oxidized states, and the peak of Mo oxide is attributed to these states. The other peaks at 530.4, 531.8, and 533.5 eV in the XPS spectrum of Mo_2_C paper are ascribed to C–Mo–O_*x*_ (O terminated), C–Mo–(OH)_*x*_ (OH terminated), and C–Mo–(OH)_*x*_–H_2_O_ads_ (OH terminated with strongly adsorbed water), respectively.^42^ In the following experiments, Mo_2_C paper was used as the cathode of the Mg–Mo_2_C batteries.

### 2.2 Electrolyte Preparation

**Figure 3:**
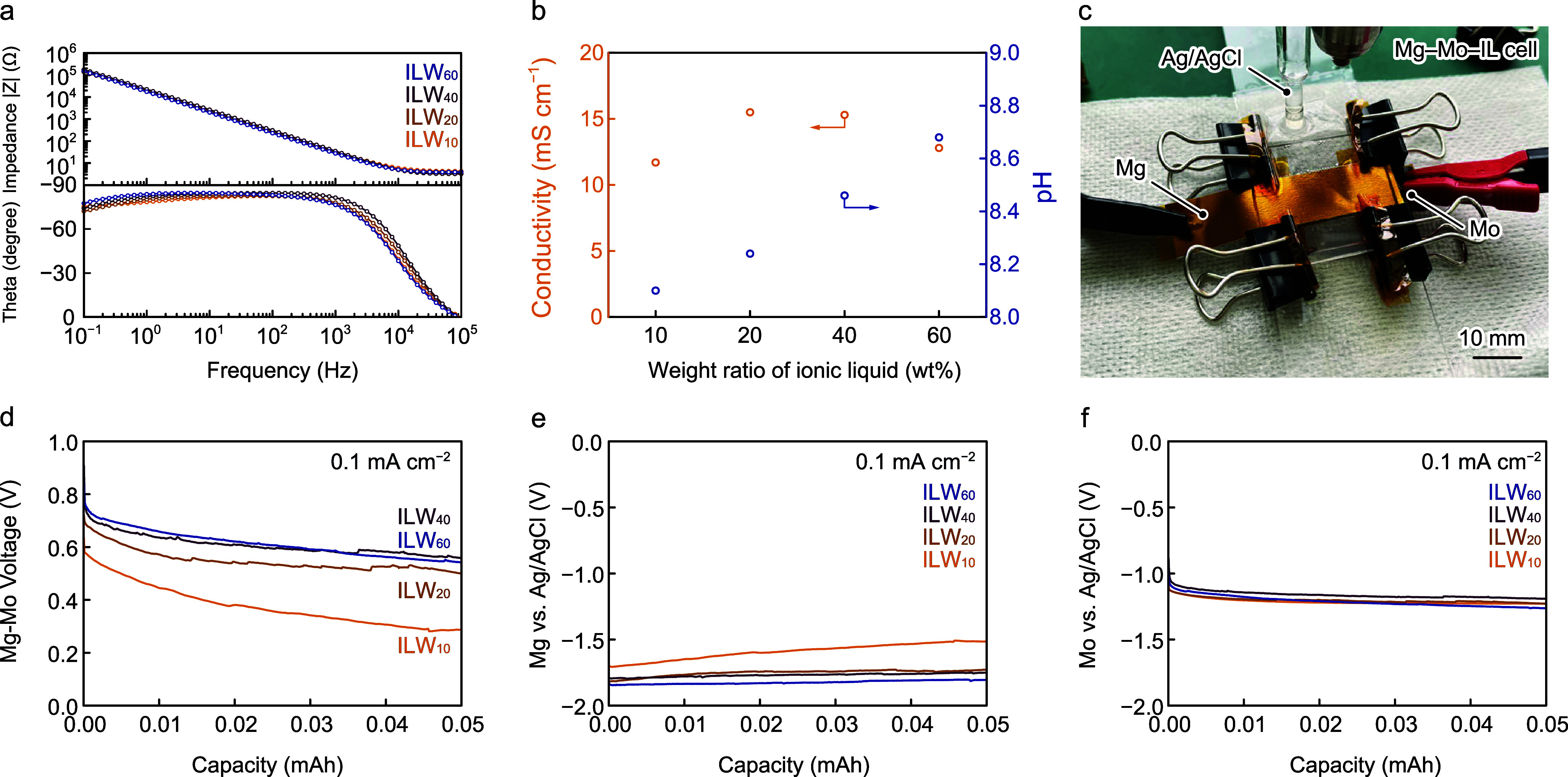
Electrochemical measurement on IL. (a) EIS spectra of ILW_10_, ILW_20_, ILW_40_, and ILW_60_ and (b) their ionic conductivity and pH at 100 kHz. (c) Optical image of Mg–Mo–IL batteries with ILWs. Discharging behavior of (d) Mg–Mo–IL cells, (e) Mg anodes, and (f) Mo cathodes with a discharge current of 0.1 mA cm^–2^ in different ILWs.

The electrolyte for Mg–Mo_2_C batteries is composed of [Ch][Lac] and DIW, and the electrochemical characteristics of the electrolyte were studied to optimize its weight ratio. Unless otherwise stated, the electrolyte is named the ILW, where the subscript number is the weight ratio of the IL. Figure 3a shows the electrochemical impedance spectroscopy (EIS) results of ILWs with IL weight ratios from 10 to 60 wt %. ILWs exhibit similar EIS curves, and the DIW content has a negligibly small influence on the impedance. ILW_20_ exhibits the highest ionic conductivity of 16 mS/cm and a pH of 8.1. With an increase in the IL weight ratios, the pH of the ILW linearly increases. Then, the performance of Mg–Mo batteries with ILW electrolytes (Mg–Mo–ILW) was studied using three-electrode cells with Mo, Mg, and Ag/AgCl as working, counter, and reference electrodes, respectively, as shown in Figure 3c. Figure 3d shows the galvanostatic discharge of the Mg–Mo–ILW batteries at 0.1 mA cm^–2^. Upon discharge, Mg–Mo–ILW_20_, Mg–Mo–ILW_40_, and Mg–Mo–ILW_60_ batteries show Mg–Mo voltages of ∼0.8 V whereas the voltage of Mg–Mo–ILW_10_ batteries is 0.6 V, which is 0.2 V lower than that of batteries with other ILWs. Figure 3e and f show the individual potentials of the anode (Mg) and cathode (Mo) vs reference electrode, respectively. Whereas the potential at the anode when ILW_20_, ILW_40_, and ILW_60_ are used as electrolytes remains stable at −1.8 V, the anode potential when ILW_10_ is used as the electrolyte increases to −1.5 V at 0.05 mAh, which shows that the Mg electrode rapidly corrodes in the water-rich ILW. The potential at the cathode remains at −1.2 V when all ILWs are used as electrolytes during discharge. Previous reports by other groups show that the following chemical reactions occur in Mg–Mo batteries:^22^Anodic reaction:
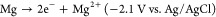
6with the following anodic side reaction:
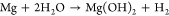
7Cathodic reactions: Oxygen reduction:
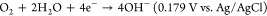
8orHydrogen evolution:
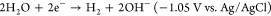
9

Compared with a previous work on Mg–Mo batteries using PBS, ILWs show low cathodic potentials and HERs as the dominant cathodic reaction. Based on the characterization, ILW_40_ was used as the electrolyte in Mg–Mo_2_C batteries.

### 2.3 Characterization of Mg–Mo_2_C Batteries Containing ILW

Three Mo_2_C electrodes were prepared using 0.25 g (Mo_2_C_0.25g_), 0.50 g (Mo_2_C_0.50g_), and 1.0 g (Mo_2_C_1.0 g_) of d-Mo_2_C. To fabricate Mo_2_C electrodes, a pristine Mo foil was cleaned with acetone, ethanol, and DIW; then, oxide on the Mo foil surface was removed using an NH_4_OH solution (Figure S4a, Supporting Information). A silicone rubber with a rectangular hole, used as a reservoir, was laminated on the pristine Mo foil, and plastic bolts and a pair of poly(tetrafluoroethylene) (PTFE) holders were used to tightly attach the rubber to the Mo foil. The suspension of d-Mo_2_C was decanted into the reservoir (Figure S4b, Supporting Information), followed by drying in the ambient air. The Mo_2_C_1.0 g_ electrode was prepared by repeatedly casting the suspension containing 0.5 g of d-Mo_2_C on the Mo foil and drying. After drying, a dark-green layer of Mo_2_C was formed on the pristine Mo foil, as shown in Figure S4c, Supporting Information. Cross-sectional SEM images show that the thicknesses of Mo_2_C layers in the Mo_2_C_0.25g_, Mo_2_C_0.5g_, and Mo_2_C_1.0g_ electrodes are 1.9, 4.9, and 6.5 μm, as shown in Figure S5a, b, and c, Supporting Information, respectively.

**Figure 4:**
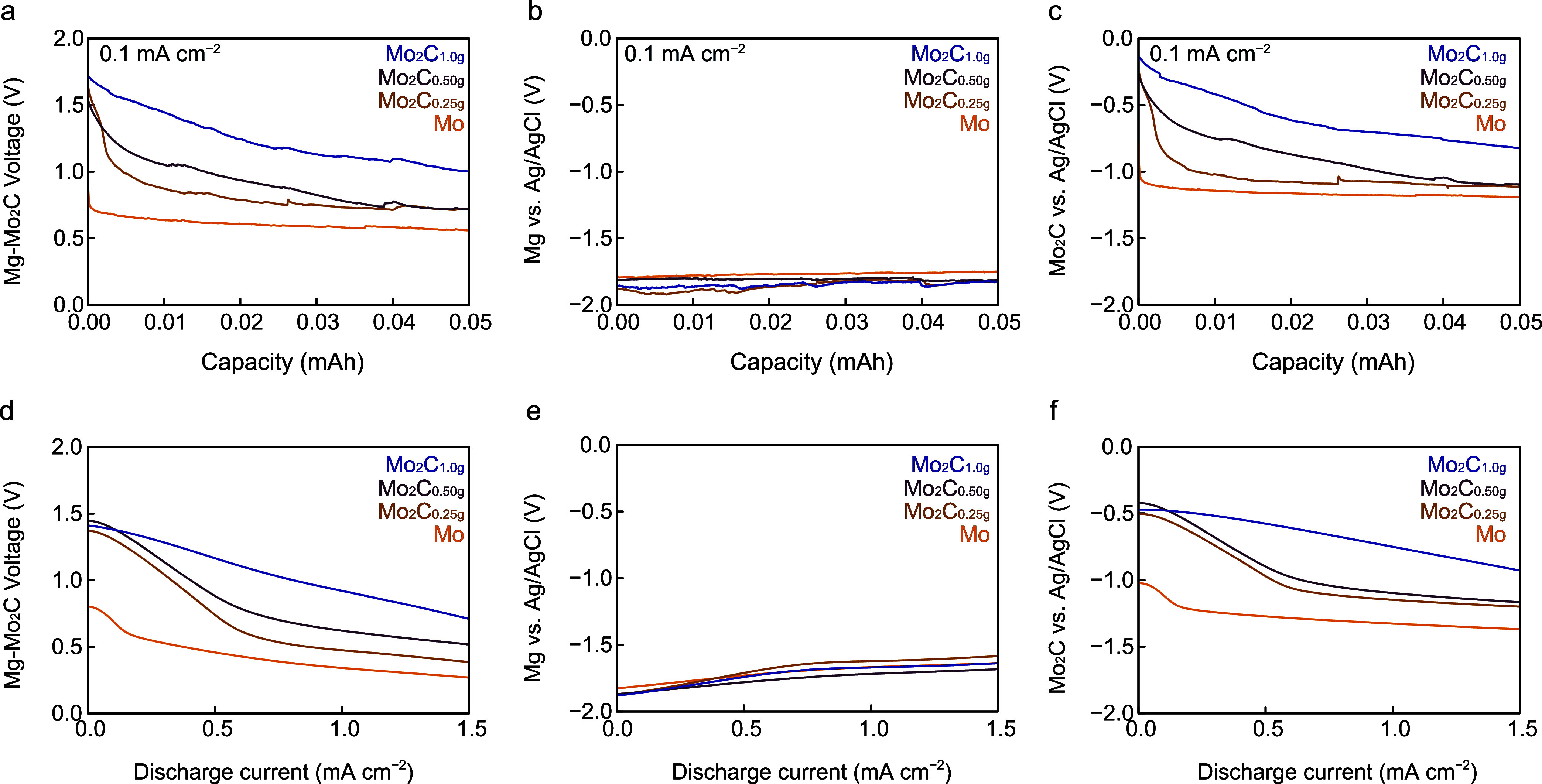
Electrochemical measurement of Mg–Mo_2_C–IL cells. Discharging behavior of (a) Mg–Mo_2_C–IL cells, (b) Mg anodes, and (c) Mo_2_C cathodes with different Mo_2_C thicknesses at a discharge current of 0.1 mA cm^–2^. Voltage vs discharge current characteristics of (d) Mg–Mo_2_C–IL cells, (e) Mg anodes, and (f) Mo_2_C cathodes at a scanning rate of 10 μA cm^–2^ s^–1^.

The performance of Mg–Mo_2_C–ILW_40_ cells was studied using three-electrode cells with Mo_2_C, Mg, and Ag/AgCl as the working, counter, and reference electrodes, respectively, where a Mg–Mo–ILW_40_ cell was characterized as a reference sample, using Mg, Mo, and IL as an anode, a cathode, and an electrolyte, respectively. Figure 4a shows the output voltage obtained using Mo, Mo_2_C_0.25g_, Mo_2_C_0.5g_, and Mo_2_C_1.0g_ cathodes during galvanostatic discharge at 0.1 mA cm^–2^. Upon discharge, voltages higher than 1.5 V are obtained for all batteries containing Mo_2_C. Mg–Mo_2_C_1.0g_–ILW_40_ batteries exhibit a voltage of 1.7 V upon discharge, which is almost twice as high as that of Mg–Mo–ILW_40_ batteries. As shown in Figure 4a, the voltage decreases as the Mg–Mo_2_C–IL batteries discharge and no plateaus are observed. The anodic potentials remain stable at −1.8 V (vs Ag/AgCl) when ILW_40_ is used as the electrolyte, as shown in Figure 4b. Whereas the Mo cathode of the Mg–Mo– ILW_40_ cell exhibits a potential of −1.0 V at 0 mAh, the potentials of Mo_2_C cathodes are higher than −0.3 V, indicating that Mo_2_C suppresses the HER. The cathodic potentials of Mo_2_C_0.25g_ and Mo_2_C_0.50g_ rapidly decrease to −1.0 V (vs Ag/AgCl) at 0.05 mAh. The potential of Mo_2_C_1.0g_ decreases to −0.7 V (vs Ag/AgCl) at 0.05 mAh. ILW_40_ is fully encapsulated with silicone rubber except for an opening to insert the Ag/AgCl electrode; therefore, insufficient oxygen supply hinders the ORR, allowing the HER to dominate in the cell. Furthermore, the decrease in potential is fast in the order Mo_2_C_0.25g_ > Mo_2_C_0.50g_ > Mo_2_C_1.0g_ electrodes, consistent with the order of their thicknesses, which indicates that ILW_40_ infiltrates the Mo_2_C layers to contact the Mo electrode and allows the HER to occur.

The anodic and cathodic reactions were analyzed, and their potentials as a function of the discharge current were studied. The output voltage of the Mg–Mo–ILW_40_ cell is 0.75 V upon discharge and decreases to 0.6 V at 0.2 mA cm^–2^, as shown in Figure 4d. With an increase in the discharge current, the voltage gradually decreases to 0.3 V at 1.5 mA cm^–2^. The Mg–Mo_2_C_1.0 g_–ILW_40_ batteries exhibit a voltage of ≈1.4 V at 0 mA cm^–2^, which is twice as high as that of the Mg–Mo–ILW_40_ cell. The voltage of cells containing Mo_2_C_0.25g_ and Mo_2_C_0.50g_ cathodes rapidly decreases at 0 mA cm^–2^. However, the voltage of the battery containing Mg–Mo_2_C_1.0 g_–ILW40 decreases more slowly than those of the cells containing Mo, Mo_2_C_0.25g_, and Mo_2_C_0.50g_ cathodes. Mg anodes maintain their potentials with a slight change of <0.3 V, as shown in Figure 4e. The reduction in the cathodic potential of Mo_2_C cathodes is shown in Figure 4f. Furthermore, at Mo cathodes, the HER becomes the dominant reaction at 0.15 mA cm^–2^. The potential of Mo_2_C_0.25g_ and Mo_2_C_0.50g_ cathodes decreases from −0.5 V to −1.0 V as the current increases from 0 to 0.5 mA cm^–2^, indicating that the HER is more dominant than the ORR. Although such switches in reactions occur at Mo_2_C_0.25g_ and Mo_2_C_0.50g_ cathodes, they suppress the HER compared with the Mo cathode. Mo_2_C_1.0 g_ cathodes show a broader curve than Mo, Mo_2_C_0.25g_, and Mo_2_C_0.50g_ cathodes, as shown in Figure 4f. The energy barriers of Mo_2_C suppress the HER and make the ORR dominant in the cell; therefore, Mo_2_C_1.0g_ cathodes were used in air batteries.

### 2.4 Solid-State Mg–Mo_2_C_1.0g_–IG Batteries

**Figure 5:**
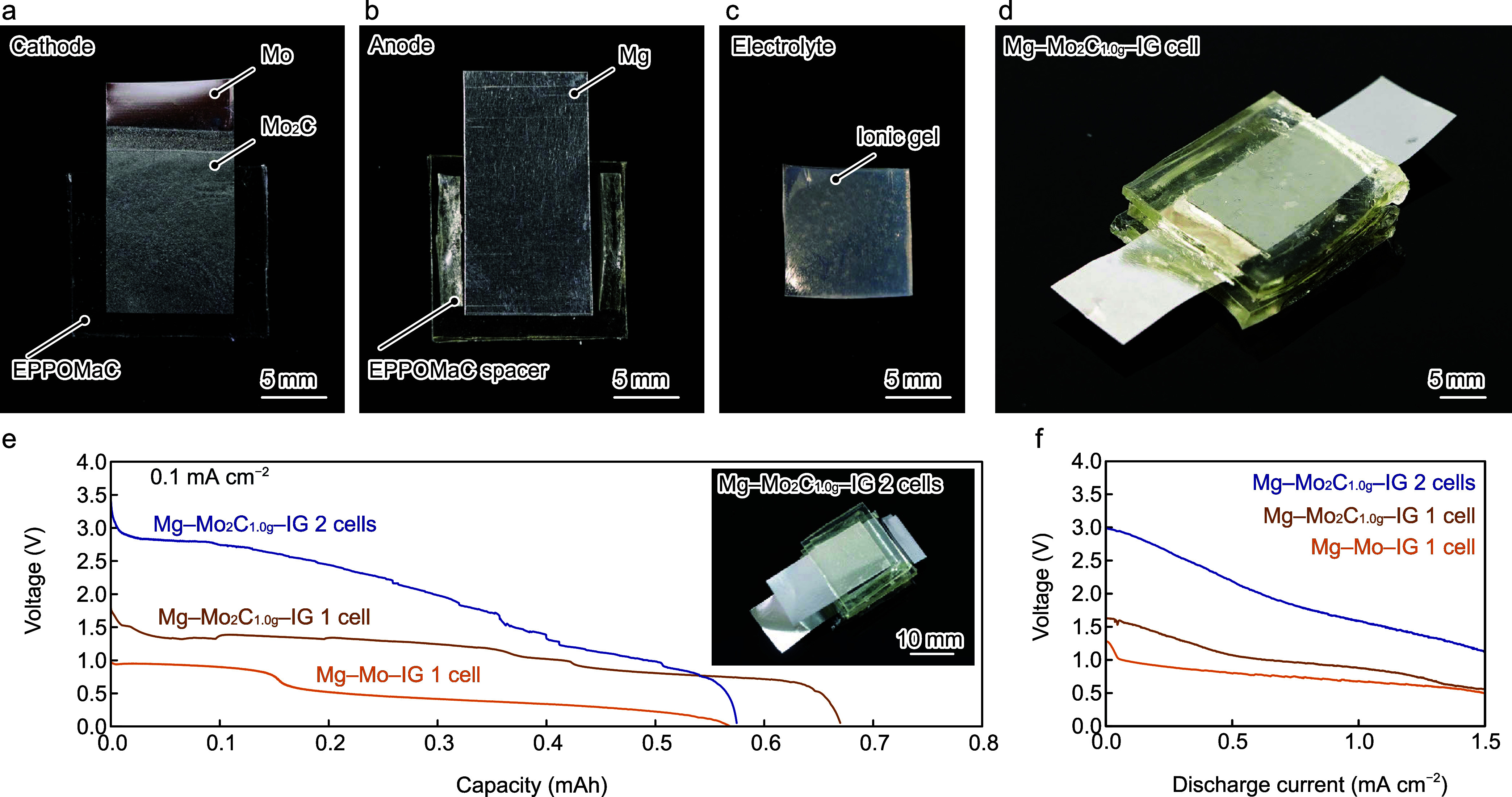
Electrochemical measurement on solid-state Mg–Mo_2_C–IG cells. Photographs of the (a) Mo_2_C_1.0g_ cathode, (b) Mg anode, (c) IG, and (d) full Mg–Mo_2_C_1.0g_–IG 1 cell. (e) Discharging behavior of the Mg–Mo_2_C_1.0g_–IG 2 cell, Mg–Mo_2_C_1.0g_–IG 1 cell, and Mg–Mo 1 cell at 0.1 mA cm^–2^. The inset photograph shows the Mg–Mo_2_C_1.0g_–IG 2 cells. (f) Voltage vs discharge current characteristics of the Mg–Mo_2_C_1.0g_–IG 2 cell, Mg–Mo_2_C_1.0g_–IG 1 cell, and Mg–Mo 1 cell at a scanning rate of 10 μA cm^–2^ s^–1^.

Solid-state Mg–Mo_2_C_1.0g_ batteries (Mg–Mo_2_C_1.0g_–IG) with an IG and a biodegradable polymer, EPPOMaC, were fabricated. An Mo_2_C_1.0g_ foil with dimensions of 20 mm × 10 mm was placed on a ≈2 mm-thick EPPOMaC sheet (Figure 5a). A Mg foil (20 × 10 mm) and a pair of EPPOMaC spacers (10 × 2 × 1 mm) were also placed on an EPPOMaC sheet (Figure 5b). EPPOMaC spacers allow the IG to supply oxygen in air for the ORR at the cathode. EPPOMaC exhibits high adhesion force, and Mo_2_C_1.0g_ and Mg foils tightly stick to EPPOMaC sheets without adhesive. ILW_40_ was dispersed in PVA with a weight ratio of DIW:ILW_40_ = 15:85 to prepare the IG, and it was cut into cubes with dimensions of 10 mm × 10 mm, as shown in Figure 5c. EIS measurements show that the conductivity of the IG is 1.2 mS cm^–2^ (Figure S6a, Supporting Information). The IG exhibits a fracture strain and Young’s modulus of 364% and 1.5 MPa, respectively (Figure S6b, Supporting Information), where values were measured in triplicate and averaged. The anode, cathode, and IG were assembled into a complete battery (Figure 5d). Figure 5e shows the discharge curves of the Mg–Mo_2_C_1.0g_–IG 1 cells, Mg–Mo_2_C_1.0g_–IG 2 cells, and Mg–Mo–IG 1 cells, where the Mg–Mo_2_C_1.0g_–IG 2 cells are two serially connected Mg–Mo_2_C_1.0g_–IG 1 cells. The Mg–Mo_2_C_1.0g_–IG 1 cell shows a voltage of 1.8 V upon discharge at 0.1 mA cm^–2^, and the discharge curve exhibits a plateau with a voltage ranging from 1.0 to 1.5 V. The voltage is 50% higher than that of the Mg–Mo–IG 1 cell with the same device configuration. While the voltage of Mg–Mo_2_C_1.0g_–IL with ILW_40_ decreases to 1.0 V at 0.05 mAh, the Mg–Mo_2_C_1.0g_–IG 1 cell with the IG retains its voltage of >1.0 V when the capacity is 0.5 mAh. The EPPOMaC spacer supplies sufficient oxygen to the electrolyte to ensure the ORR is dominant at the cathode. Furthermore, the IG contacts the Mo_2_C layer alone, preventing ILW_40_ from infiltrating into the Mo layer, which results in such a high operating voltage. The Mg–Mo_2_C_1.0g_–IG 2 cells exhibit a voltage of 3.3 V at 0 mAh, which is approximately twice as high as that of the Mg–Mo_2_C_1.0g_–IG 1 cell, and the voltage remains at 2.8 V when the capacity is 0.1 mAh. The voltage of the Mg–Mo_2_C_1.0g_–IG 1 cell decreases at a capacity of 0.1 mAh, probably due to the decrease in the contact area between the IG and Mo_2_C layer due to volume reduction associated with water evaporation in the IG. The output voltage of the Mg–Mo_2_C_1.0g_–IG 1 cell, 2 cells, and Mg–Mo–IG 1cell as a function of the discharge current was studied. As shown in Figure 5e, the Mg–Mo_2_C_1.0g_–IG 1 cell exhibits a voltage of 1.6 V at 0 mA cm^–2^, which gradually decreases to 0.7 V at 1.5 mA cm^–2^. In the small-current regime of <1.0 mA cm^–2^, the voltage of the Mg–Mo_2_C_1.0g_–IG 1 cell is higher than that of the Mg–Mo–IG 1 cell, but it becomes as small as that of the Mg–Mo–IG 1 cell at 1.3 mA cm^–2^, which indicates that the HER is dominant in the large-current regime. The voltage of the Mg–Mo_2_C_1.0g_–IG 2 cells is twice as high as that of the Mg–Mo_2_C_1.0g_–IG 1 cell with the same discharge current, and the maximum powers of the Mg–Mo_2_C_1.0g_–IG 1 and 2 cells are 0.92 and 1.7 mW cm^–2^, respectively. Compared with previous studies on the transient primary batteries as summarized in Table 1, the Mg–Mo_2_C_1.0g_–IG 1 cell exhibits operating voltages higher than those of Mg–X (X = Fe, W, and Mo) and Zn–Mo batteries and comparable with those of Mg–Au, Mg–MoO_3_, and Mg–I_2_/C batteries. Furthermore, the maximum power of the Mg–Mo_2_C_1.0g_–IG 1 cell is the highest among those of reported transient primary batteries. The Mg–Mo_2_C_1.0g_–IG battery suffers from low capacity due to the reduction in contact area during discharge due to the shrinkage of the IG during water evaporation; however, an optimized design that hinders evaporation can yield high capacities.

**Table 1:** Comparison of Materials Used to Fabricate Transient Primary Batteries and the Obtained Operating Voltages, Maximum Powers, and Capacities Reported in Recent Studies of Transient Primary Batteries

no.	electrode	electrolyte	operating voltage	maximum power	capacity	year	ref
1	Mg–Mo_2_C (anode–cathode)	ionic gel [Ch][Lac]:DIW:PVA = 34:51:15	1.4 V	0.92 mW cm^–2^	0.57 mAh cm^−2^ at 0.1 mA cm^–2^	2023	this work
2	Mg–Mo, W, or Fe (anode–cathode)	phosphate-buffered saline (PBS) solution	0.45 V		276 mAh g^–1^ at 0.1 mA cm^–2^(Mg/Mo)	2014	ref^22^
3	Mg–Au (anode–cathode)	hydrogel (silk fibroin aqueous solution 7.5 wt %)	1.0 V	8.7 μW cm^–2^	0.06 mAh cm^–2^ at 10 μA cm^–2^(unsealed 1.43 mAh cm^–2^)	2017	ref^23^
4	Mg–MoO_3_(anode–cathode)	hydrogel (sodium alginate), PBS solution	1.6 V	0.27 mW cm^–2^	6.5 mAh cm^–2^	2018	ref^29^
5	Mg–MoO_3_(anode–cathode)	hydrogel (sodium alginate), PBS solution	1.5 V	0.196 mW cm^–2^	1.72 mAh cm^–2^ at 45 μA cm^–2^	2022	ref^30^
6	Mg–I_2_/C (anode–cathode)	PBS/ChCl urea-based ILs (anolyte/catholyte)	1.8 V	≈0.7 mW cm^–2^	3.9 mAh cm^–2^ at 0.4 mA cm^–2^	2022	ref^24^
7	Zn–Mo(anode–cathode)	0.9 wt % NaCl saline or hydrogel	0.6 V	6 μW cm^–2^	1,596 μWh at 5 μA cm^–2^, 1728 μWh at 10 μA cm^–2^	2023	ref^31^

### 2.5 Wireless Sensing Using Mg–Mo_2_C_1.0g_–IG Batteries

**Figure 6:**
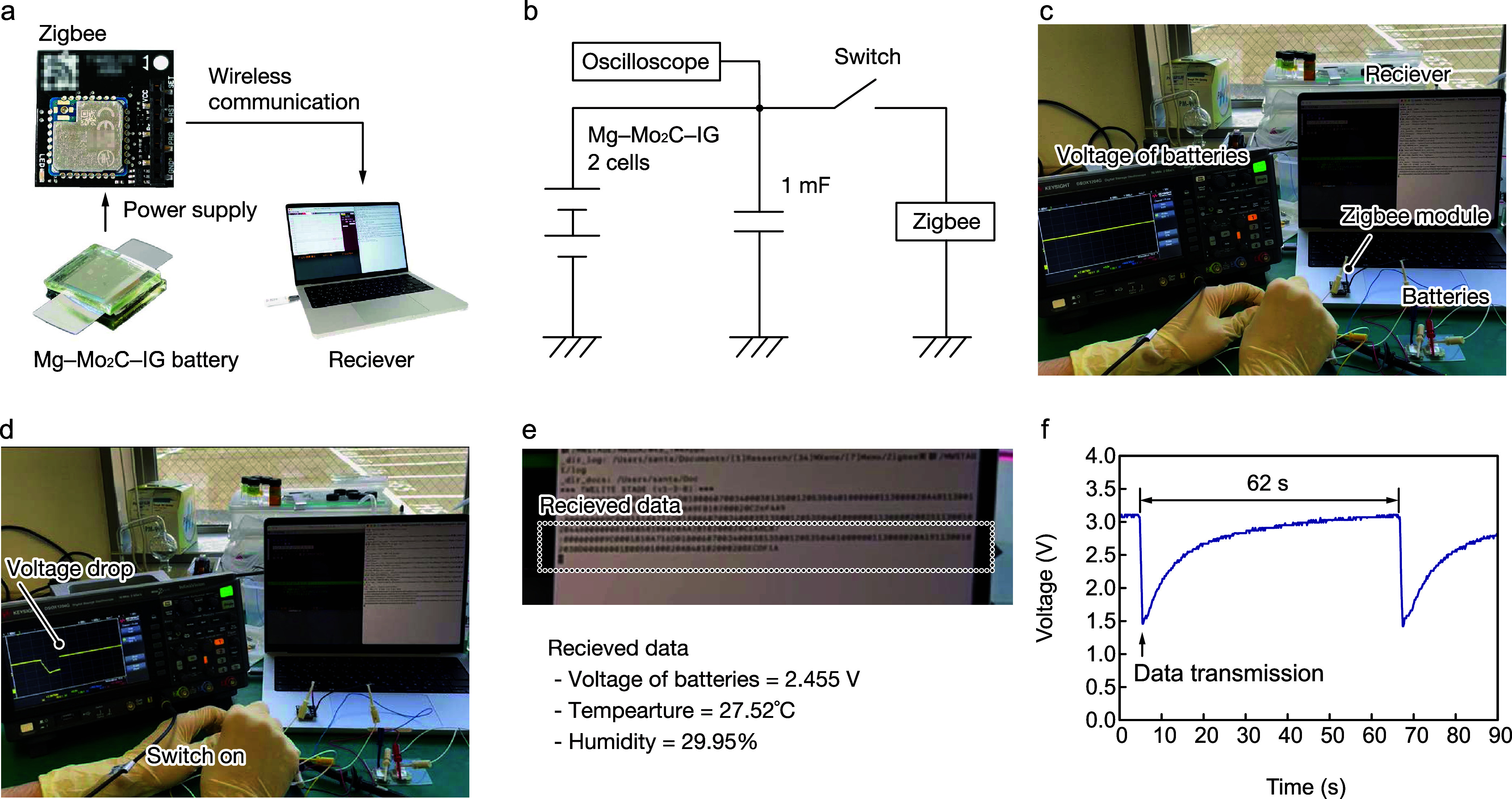
Demonstration of wireless communication powered by Mg–Mo_2_C_1.0g_–IG 2 cells. Schematics of the (a) wireless sensing and (b) electrical circuits for a sensing node. Photograph of an experimental bed and change in the voltage of the Mg–Mo_2_C_1.0g_–IG 2 cells (c) before and (d) after wireless communication. (e) Photograph of the received data from the sensing node. (f) Recovery of the voltage at 1 mF.

The voltage of the Mg–Mo_2_C_1.0g_–IG 2 cells is sufficiently high to operate wireless sensor modules including Zigbee, Bluetooth, and LoRaWAN for environmental sensing. To study the practical application of Mg–Mo_2_C_1.0g_–IG batteries, wireless sensing using a Zigbee module was performed, as shown in Figure 6a. The Zigbee module, powered by the Mg–Mo_2_C_1.0g_–IG 2 cells, sent temperature, humidity, and operating voltage signals to a laptop with a receiver module. The Zigbee module was connected with the Mg–Mo_2_C_1.0g_–IG 2 cells and the 1 mF capacitor via a mechanical switch (Figure 6b); it sent data when the switch was turned on. An oscilloscope monitored the voltage at the Mg–Mo_2_C_1.0g_–IG 2 cells during the experiment. Figure 6c shows a photograph of the experimental bed. The voltage of the Mg–Mo_2_C_1.0g_–IG 2 cells decreases from 3.1 to 1.5 V when the mechanical switch is turned on (Figure 6d), and the laptop receives data sent by the sensor module, as shown in Figure 6e and Movie S1, Supporting Information, where the temperature, humidity, and operating voltage were 27.53 °C, 29.95%, and 2.455 V, respectively. The Mg–Mo_2_C_1.0 g_–IG 2 cells cannot sufficiently power the module; therefore, the voltage reverts to its initial value after 62 s. The output power can be increased by optimizing the structure of the Mg–Mo_2_C_1.0g_–IG 2 cells to supply oxygen to the IG and increasing the active surface by using a kirigami structure, which can shorten data transmission intervals.

### 2.6 Dissolution Test of Mg–Mo_2_C_1.0g_–IG Batteries

**Figure 7:**
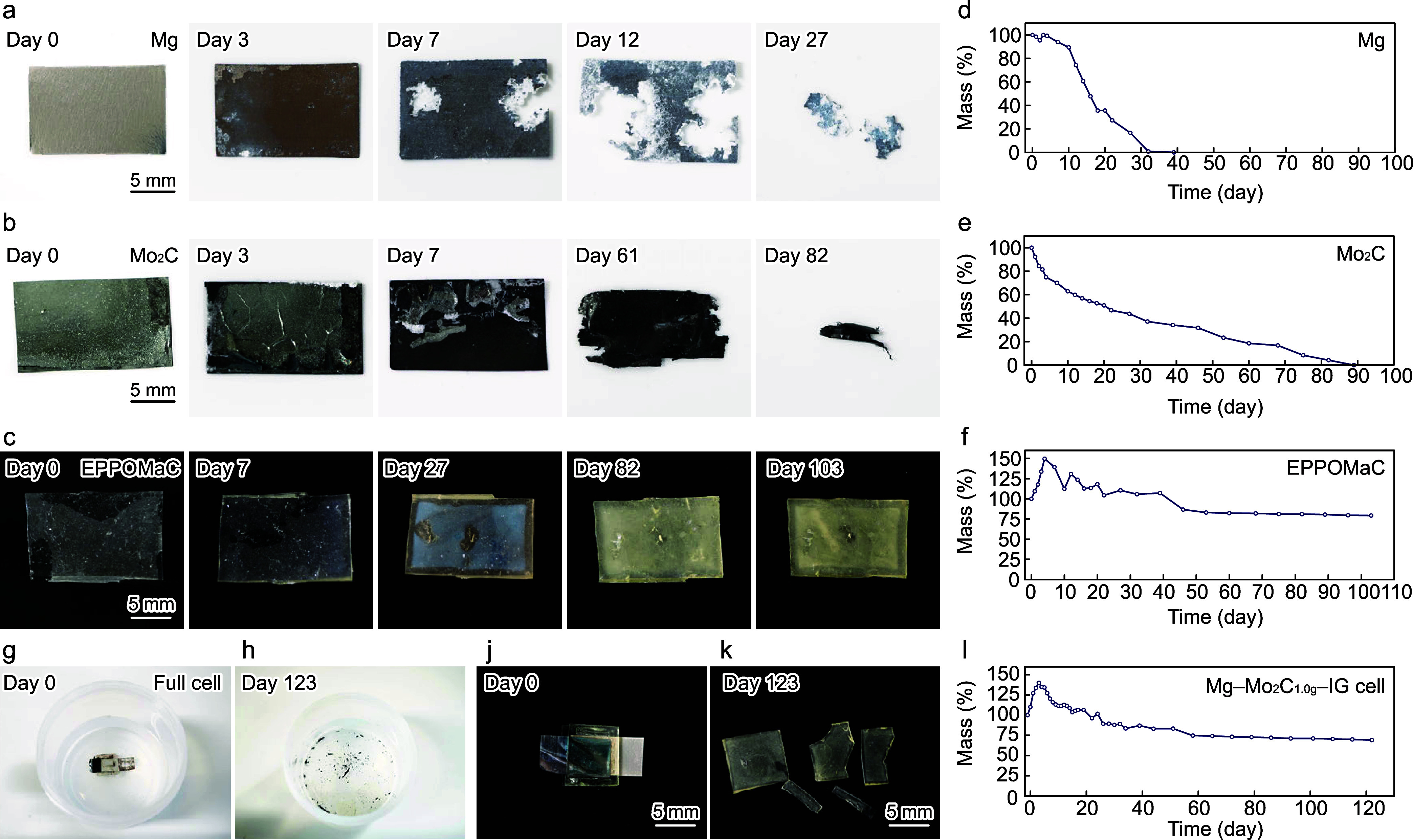
Degradation of the Mg–Mo_2_C_1.0g_–IG cell and its individual components. Time-lapse images and change in the mass of the (a, d) Mg, (b, e) Mo_2_C, (c, f) EPPOMaC, and (g–l) Mg–Mo_2_C_1.0g_–IG 1 cell during degradation in 100 mL of a 10 mM PBS solution at 37 °C.

The transient behavior of individual components and cell were investigated by soaking them in 100 mL of a 10 mM PBS solution at 37 °C. Figure 7a, b, and c show the time-lapse images of Mg, Mo_2_C_1.0g_, and EPPOMaC, respectively, and the corresponding changes in mass are shown in Figure 7d, e, and f, respectively. The change in mass *M*_c_ is defined as the ratio of mass *M* with respect to initial mass *M*_0_, expressed by the following equation:
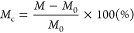
10

The Mg anode has a silver-gray color that turns brown between days 0 and 3 of the dissolution test. The Mg foil starts to decompose at day 7, forming holes and white parts of Mg(OH)_2_; its mass simultaneously starts to decrease. Most of the surface of the Mg foil is covered with a white layer of Mg(OH)_2_, and the degradation spreads from holes by day 12. Leaving only small fragments left, the Mg foil almost completely dissolves in the PBS solution by day 27 and fully dissolves by day 39. As for Mo_2_C cathodes, Mo_2_C, which tightly adheres to the Mo foil (day 0), starts to detach on day 3. Most Mo_2_C peeled off by day 7 and dispersed into the PBS solution. Its mass decreased rapidly within the first 10 days of the dissolution test, probably due to the dissolution of Mo_2_C. The Mo foil gradually dissolves into the PBS solution, as previously reported,^32^ and its mass linearly decreases after the dissolution of Mo_2_C. The edges of the Mo foil start to decompose on day 61, and the foil breaks down into fragments by day 82. PBS, finally, breaks down the Mo foil into small pieces on day 89. However, EPPOMaC is stable in PBS solution and no changes in its appearance are observed until day 7. Notably, EPPOMaC swells in PBS solution and its mass increases by 50% by day 4. Although the materials were vacuum annealed at 40 °C before weight measurements, some PBS remains in EPPOMaC and the fluctuation of its mass can be attributed to residual moisture in the polymer. The residual moisture changes the color of EPPOMaC to an opaque white by day 27, as shown in the photograph. Whereas the polymer poly(1,8-octanediol-*co*-citrates) (POC) easily decomposes in DIW at 65 °C via hydrolysis,^19^ the ester bonds in EPPOMaC make it highly stable in the PBS solution. Whereas its color changes to opaque yellow by day 82, EPPOMaC remains intact on day 103 with a 20% decrease in mass. Although EPPOMaC does not completely degrade during the test because of its chemically strong ester bonding, materials, i.e., 1,8-octanediol, citric acid, and maleic anhydride, can biodegrade, and previous works have reported its in vivo degradation behavior.^11,48^ Therefore, EPPOMaC is a promising candidate for Mg–Mo_2_C_1.0g_–IG cells. The Mg–Mo_2_C_1.0g_–IG 1 cell was immersed in a PBS solution to investigate its transient behavior. Figure 7g and h shows the Mg–Mo_2_C_1.0g_–IG 1 cell on days 0 and 123, respectively. The Mg electrode completely dissolves in the PBS solution, and Mo decomposes into small fragments. The individual components are tightly attached to form the cell, as shown in Figure 7j, and when the cell decomposes, it leaves behind Mo fragments and EPPOMaC, as shown in Figure 7k. The change in mass was monitored during the dissolution test. As shown in Figure 7l, the mass increases by ≈40% on day 5 due to the swelling of EPPOMaC. Henceforth, the mass decreases to 69% by day 123.

## 3 Conclusions

A biodegradable primary battery was developed using Mo_2_C MXene and a bioderived IL. Mo_2_C exhibits energy barriers for the HER, which can suppress an undesired HER at the cathode. A Mo electrode coated with an Mo_2_C layer was used as cathodes to increase the output voltage of the Mg–Mo_2_C_1.0g_–IG cells to 1.4 V, which is twice as high as Mo cathodes. The Mg–Mo_2_C_1.0g_–IG 1 cell shows a maximum power of 0.92 mW cm^–2^, and with such characteristics, the Mg–Mo_2_C_1.0g_–IG 2 cells can operate as a wireless sensor module. Degradation tests indicate that Mg and Mo electrodes dissolve in the PBS solution within 39 and 89 days, respectively. EPPOMaC does not dissolve in the PBS solution because of its strong ester bonding, which can yield the stable operation of batteries. Future applications of power sources based on transient primary batteries include biomedical, wearable, and environmentally benign devices that decompose after use.

## 4 Experimental Section

### 4.1 Synthesis of MXene

Pristine Mo_2_C powders (product ID: 399531, Sigma-Aldrich) and Ga flakes (GAE03PB, Kojundo Chemical Lab., Co., Ltd.) were heated at 70 °C and homogeneously mixed using an agate mortar in a molar ratio of Ga:Mo_2_C = 8:1. The mixture was vacuum encapsulated in a quartz tube and annealed at 850 °C for 48 h to obtain a Mo_2_Ga_2_C powder. The Mo_2_Ga_2_C powder was soaked in a 37 wt % HCl solution for 24 h to remove unreacted Ga. 2.5 g of the Mo_2_Ga_2_C powder and 80 mL of a 49% HF solution were sealed into a perfluoroalkoxy alkane (PFA) container, which was stirred and heated at 55 °C for 160 h to remove the layer of Ga from the MAX phase. 1 g of the Mo_2_CT_*x*_ powder was dispersed in 4 mL of TBAOH (product ID: 86863, Sigma-Aldrich), followed by ultrasonication for 1 h to allow TBAOH to intercalate into the interlayers of Mo_2_CT_*x*_. The Mo_2_CT_*x*_ powder was washed three times with ethanol, and 1 g of the Mo_2_CT_*x*_ powder was dispersed in 4 mL of DIW. Then, the dispersion solution was ultrasonicated for 1 h and centrifuged at 3500 rpm for 30 min to afford a suspension of d-Mo_2_C. An 8 mL aliquot of the d-Mo_2_C suspension was vacuum filtered onto nanoporous polypropylene membranes (Celgard 3501, CELGARD), and dark-green Mo_2_C paper was obtained after drying. Mo_2_C cathodes were prepared by casting the suspensions using 0.25 and 0.5 g of d-Mo_2_C on a Mo foil, followed by drying in ambient air. The casting process was repeated using the suspension prepared using 0.5 g of d-Mo_2_C and drying to form Mo_2_C_1.0 g_ cathodes.

### 4.2 Characterization of Mo_2_C Paper

The cross-sectional images and material compositions of Mo_2_C paper were obtained via SEM (SU-70, Hitachi High-Technologies Corporation) and EDX (Aztec Energy X-Max, Oxford Instruments), respectively. The images of the single layer of d-Mo_2_C and cross-sectional images of Mo_2_Ga_2_C were obtained via TEM (Titan^3^ 60-300 Double Corrector, Thermo Fisher Scientific) with an acceleration voltage of 200 kV and a STEM detector with an acceptance HAADF angle of 79–200 mrad. The corresponding EDX mappings of Mo_2_Ga_2_C were obtained by using an energy-dispersive X-ray spectrometer (Super-X, Thermo Fisher Scientific). A Bruker D8 ADVANCE instrument (Cu Kα, 0.15418 nm) was used to obtain XRD patterns with an accelerating voltage and currents of 40 kV and 40 mA, respectively. XPS spectra were obtained using an X-ray spectrometer (AXIS-ULTRA, Shimadzu Corporation, Kyoto, Japan).

### 4.3 Electrochemical Characterization on ILWs and Batteries

EIS measurements of ILWs were performed according to our previously reported method.^21,34^ Batteries were characterized by using a potentiostat and a galvanostat (PGSTAT204, Autolab) with an EIS module (FRA32M, Autolab). For the three-electrode cell measurements, a Ag/AgCl electrode filled with 3 M NaCl (RE-1B, BAS) was used as the reference electrode. The anode–cathode spacing, maintained using a silicone spacer, was ≈1 mm, and polyimide tapes covered the back and front sides of the electrodes to ensure the surface area was 10 × 10 mm. The pH of the ILWs was measured using a pH meter (LAQUAtwin pH-33, HORIBA Scientific).

### 4.4 Synthesis of EPPOMaC

1,8-Octanediol (>99.0%, product ID: O0024, Tokyo Chemical Industry Co., Ltd.), citric acid (ACS reagent, ≥99.5%, product ID: 251275, Sigma-Aldrich), and maleic anhydride (>99.0%, product ID: M0005, Tokyo Chemical Industry Co., Ltd.) were mixed in a four-neck round-bottom flask in a molar ratio of 5:2:3, with the total mass of 50 g. Materials were initially heated at 160 °C and stirred in a nitrogen atmosphere. When materials melted to form a transparent and viscous liquid, the temperature was decreased to 140 °C, followed by further stirring for 3 h. The melt was cooled to room temperature and dissolved in 80 mL of ethanol. To remove monomers and oligomers, the solution was purified via dropwise precipitation into 2 L of DIW. The precipitate was carefully decanted into a polypropylene bottle, followed by vacuum annealing at 50 °C and 1 Pa for 48 h to remove residual ethanol and moisture. Vacuum annealing yielded a transparent POMaC precursor (Figure S7a, Supporting Information). Polymerization was performed by dissolving 0.1 g of the photoinitiator 2-hydroxy-4′-(2-hydroxyethoxy)-2-methylpropiophenone, in 1 mL of ethanol. The solution and 5 g of the POMaC precursor were decanted into a PTFE vessel (Figure S7b, Supporting Information), followed by mixing for 10 min. The content was vacuum annealed at 50 °C and 1 Pa to remove ethanol until bubbling stopped (≈48 h). A 365 nm UV light was used to cure POMaC (Figure S7c, Supporting Information). POMaC was heated in an oven at 100 °C for 8 h to cross-link ester bonds and complete polymerization, which yielded pale-yellow EPPOMaC with a thickness of ≈1 mm (Figure S7d, Supporting Information).

### 4.5 Dissolution of Mg–Mo_2_C Batteries

Salts NaCl (8.0 g), KCl (0.2 g), Na_2_HPO_4_·12H_2_O (2.9 g), and KH_2_PO_4_ (2.9 g) were mixed in DIW (1 L) to prepare a 10 mM PBS solution. The individual components, i.e., Mg, Mo, and EPPOMaC, with dimensions of 10 × 15 mm, and Mg–Mo_2_C_1.0 g_–IG batteries were immersed in 100 mL of PBS, which was stored in an oven at 37 °C. The solutions were refreshed daily.

## Data Availability

Details of the experiments are available within the article and the Supporting Information.

## Supporting Information Available

Synthesis and characterization of Mo_2_Ga_2_C; standard crystal plane of Mo_2_Ga_2_C; standard crystal plane of 1T-d-Mo_2_C; fabrication of the Mo_2_C cathode; thickness of Mo_2_C layers; characteristics of the IG; and synthesis of EPPOMaC (PDF)
Laptop receiving data sent by the sensor module (MP4)
