# Supplementary material for: Biodegradable Mg–Mo2C MXene Air Batteries for Transient Energy Storage
Source: ACS Appl Mater Interfaces. 2024 Mar 18;16(12):14759–69. doi: 10.1021/acsami.3c17692 (PMC10982942; doi:10.1021/acsami.3c17692)
Supplement: Supplementary file 1 — am3c17692_si_001.pdf [file am3c17692_si_001.pdf]

Supporting Information

Biodegradable Mg-Mo_2C MXene Air Batteries for Transient Energy Storage

Shunsuke Yamada*

Room 113, Building No. A15, Area A01, 6-6-01 Aoba, Aramakiaza, Aobaku, Sendaishi, Miyagi, 980-8579, Japan

Corresponding Author

Shunsuke Yamada

Room 113, Building No. A15, Area A016-6-01 Aoba, Aramakiaza, Aobaku, Sendaishi, Miyagi, 980-8579, Japan.

orcid.org/0000-0002-9084-2070;

E-mail: santa@tohoku.ac.jp

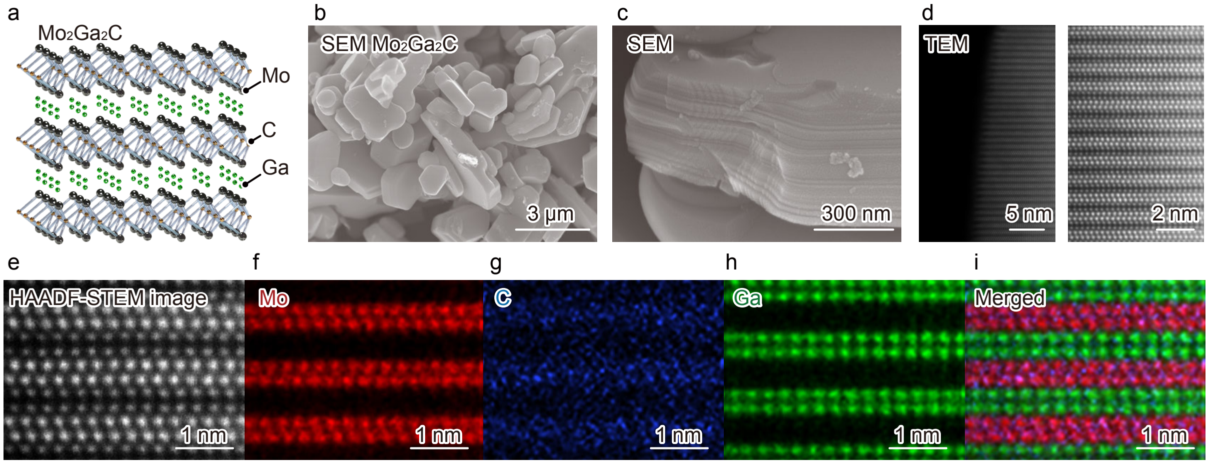

Figure S1. Synthesis and characterization of Mo_2Ga_2C. (a) Schematic and (b) SEM image of Mo_2Ga_2C. (c) SEM image showing the layered profile at the lateral face of a Mo_2Ga_2C flake. (d) HAADF-STEM image (left) of the uniform layered structure of Mo_2Ga_2C and its magnified view (right). (e) HAADF-TEM image and corresponding EDX mapping images of (f) Mo, (g) C, (h) Ga, and (i) merged.

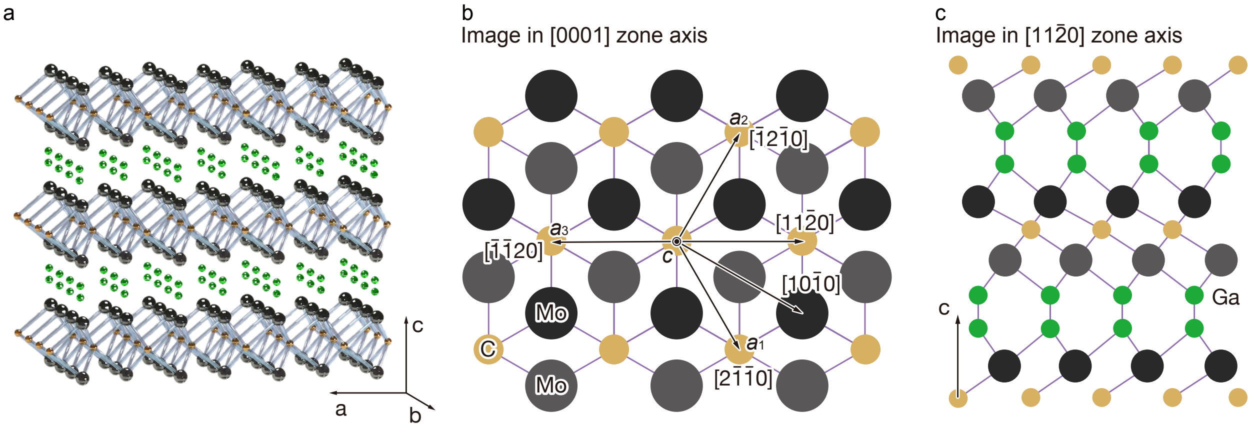

Figure S2. Standard crystal plane of Mo_2Ga_2C. (a) Schematic illustration of Mo_2Ga_2C. Images in (b) [0001] and (c) [11$\bar{2}$0] zone axes.

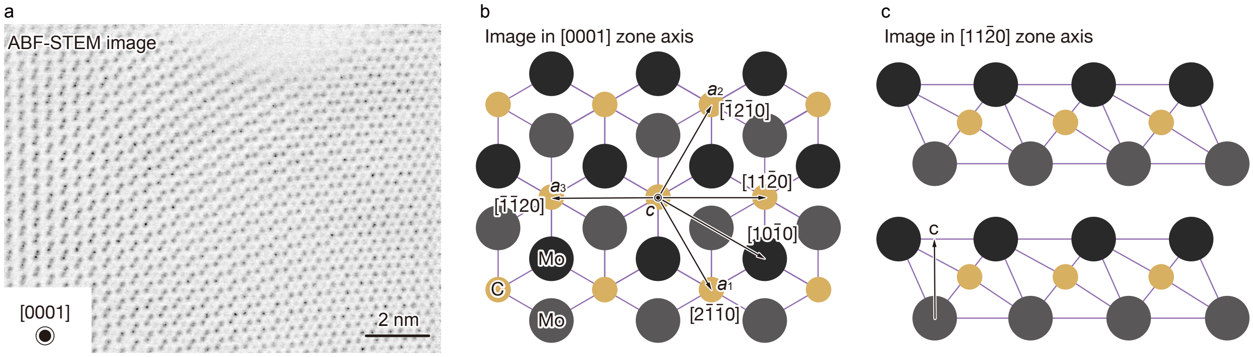

Figure S3. Standard crystal plane of 1T-d-Mo_2C. (a) An ABF-STEM image of d-Mo_2C. Images of 1T-d-Mo_2C in (b) [0001] and (c) [$11\bar{2}0$] zone axes.

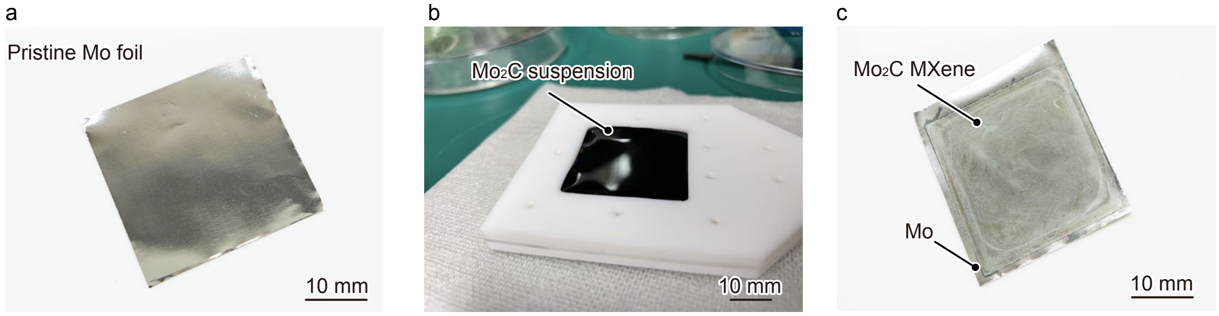

Figure S4. Fabrication of the Mo_2C cathode. (a) Photograph of a pristine Mo foil after cleaning. (b) PTFE holders and a silicone rubber were used as a reservoir, and the d-Mo_2C suspension was casted on the pristine Mo foil. (c) Photograph of the Mo foil coated with a Mo_2C layer after drying.

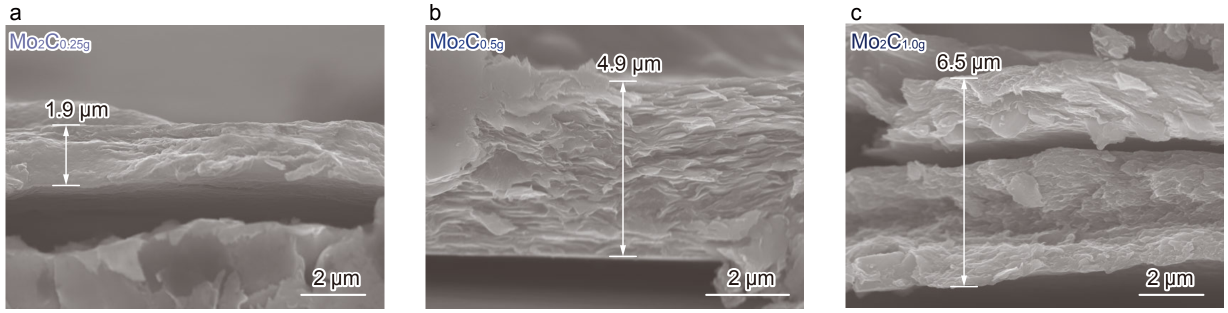

Figure S5. Thickness of Mo_2C layers. Cross-sectional SEM images of Mo_2C cathodes prepared using (a) 0.25, (b) 0.5, and (c) 1.0 g of Mo_2CT_x.

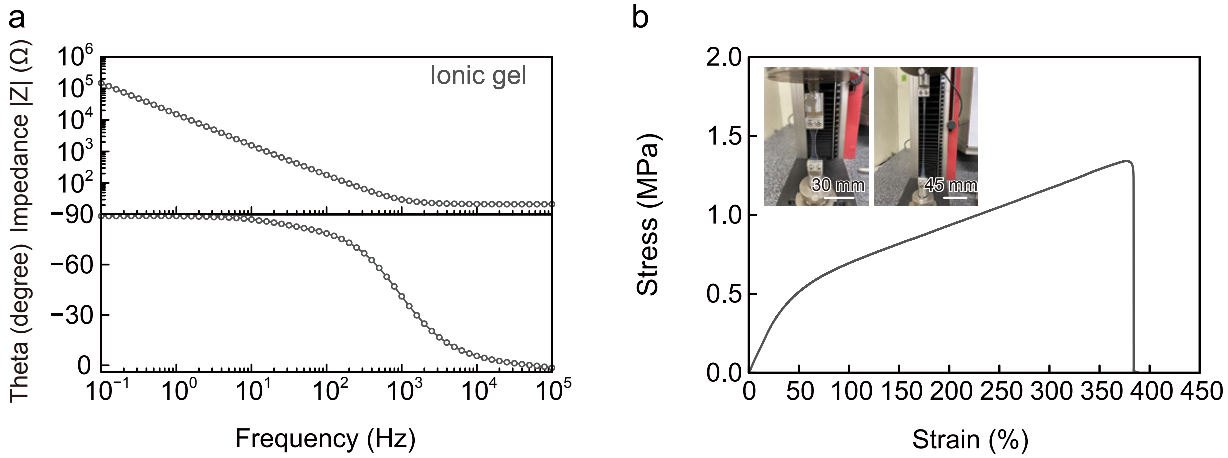

Figure S6. Characteristics of the IG. (a) EIS spectra of the IG composed of 85 wt% ionic solution (deionized water:[Ch][Lac] = 4:6) and 15 wt% PVA. (b) Tensile stress–strain curve of the IG and (inset) photographs of the IG before and after stretching.

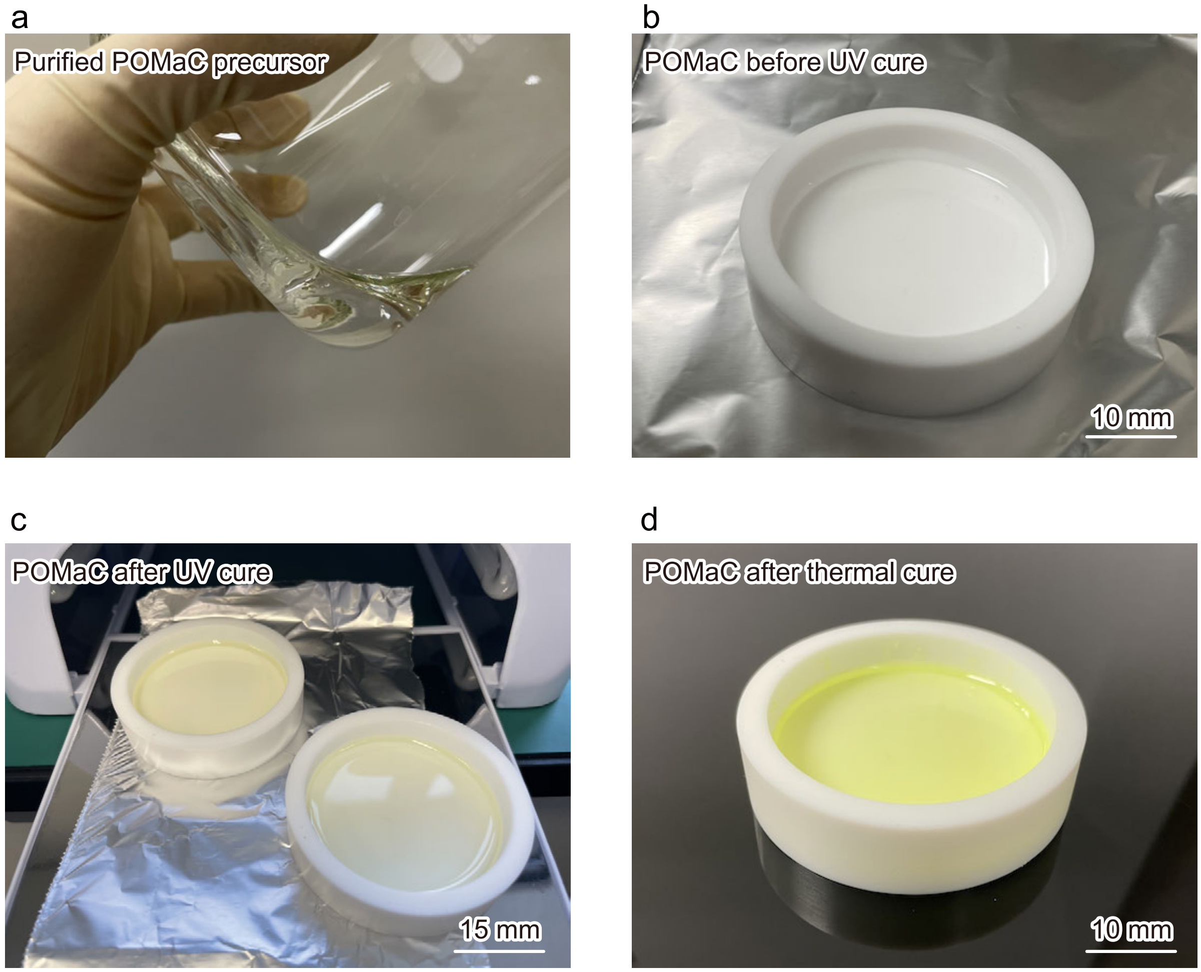

Figure S7. Synthesis of EPPOMaC. (a) Photograph of the POMaC precursor purified via dropwise precipitation. (b) The POMaC precursor mixed with a photoinitiator, 2-hydroxy-4′-(2-hydroxyethoxy)-2-methylpropiophenone was decanted into a PTFE container. (c) POMaC turned light yellow after UV curing. (d) Heat curing afforded pale yellow and sticky EPPOMaC.
